# Protocol for a scoping review of potential vaccine candidates predicted by VaxiJen for different viral pathogens between 2017–2021

**DOI:** 10.1186/s13643-022-02121-0

**Published:** 2022-12-30

**Authors:** Zakia Salod, Ozayr Mahomed

**Affiliations:** Discipline of Public Health Medicine, School of Nursing and Public Health, University of KwaZulu-Natal, Durban, KwaZulu-Natal South Africa

**Keywords:** VaxiJen, Viruses, Reverse vaccinology, Antigens, Vaccinology, Scoping review, Protocol

## Abstract

**Background:**

Vaccination is essential for the prevention of infectious diseases and has helped to reduce disease-related mortality, such as pneumonia. However, traditional vaccine development is time-consuming and risky. Reverse vaccinology (RV) is a promising alternative to developing vaccines based on the in silico discovery of antigens, often termed ‘potential vaccine candidates’ (PVCs), using a pathogen’s proteome. RV prediction technologies, such as VaxiJen (founded in 2007), are used to take the first step toward vaccine development. VaxiJen is used by researchers to identify PVCs for various diseases. A 10-year review of these PVCs was published in 2017. There has since been no review of viral PVCs predicted by VaxiJen from 2017 to 2021. The proposed scoping review aims to address this gap.

**Methods:**

This protocol is reported according to the Preferred Reporting Items for Systematic Reviews and Meta-Analyses Protocols (PRISMA-P) 2015 checklist. The review will employ Arksey and O’Malley’s five-stage methodological framework, which was later enhanced by Levac et al. and the Joanna Briggs Institute (JBI). The PRISMA extension for Scoping Reviews (PRISMA-ScR) reporting guideline will be utilized with this framework. PubMed, Scopus, Web of Science, EBSCOhost, and ProQuest One Academic will be searched using the term ‘vaxijen’. The inclusion criteria will be English-only full-text original articles published in peer-reviewed journals and unpublished papers from 2017 to 2021. Rayyan will be used to deduplicate, screen titles and abstracts of articles. The articles’ full texts will be examined. The data will be extracted using Microsoft Excel. Using a data charting form, data will be sifted and organized by key categories and themes.

**Discussion:**

This protocol was submitted for publication and went through an extensive peer review process. The review has implications for novel vaccine development against various viruses. The key limitation of this study is language bias due to the selection of English-only papers because of limited resources. This study will not require ethical clearance since it will use secondary data and will not include patients. Nevertheless, this research is part of a larger project that was submitted for ethical consideration to the Biomedical Research Ethics Committee of the University of KwaZulu-Natal in South Africa. This study’s findings will be published in a peer-reviewed journal and provided to relevant stakeholders.

**Systematic review registration:**

Open Science Framework (OSF): https://osf.io/ht8wr

**Supplementary Information:**

The online version contains supplementary material available at 10.1186/s13643-022-02121-0.

## Background

Vaccination is essential for the prevention of infectious diseases and has helped to reduce disease-related mortality, such as pneumonia. The World Health Organization (WHO) reports that immunization prevents 2–3 million deaths annually of diseases, such as influenza and measles [1]. In the last two centuries, vaccination has resulted in (i) the global elimination of smallpox in 1980 [2], and the near-eradication of other diseases such as polio and measles [3], and (ii) a 97% reduction in mortality from diseases including pneumonia, measles, mumps, rubella, and hepatitis B [4]. The human immunodeficiency virus (HIV), hepatitis C, herpes simplex, cytomegalovirus, and rhinovirus are among the diseases for which there is presently no effective vaccine [5].

The traditional vaccine development paradigm used to develop the above vaccines involves isolating, inactivating, and injecting a pathogen [6–8]. This method is (i) time-consuming, as the process takes five to 15 years; (ii) risky, as the pathogen must be grown in a laboratory; and (iii) confined to protective antigens expressed in vitro [9]. Reverse vaccinology (RV), an alternative approach to developing vaccines, may be able to address these challenges and aid in the creation of novel vaccines for a variety of illnesses [9].

RV is a modern method of screening a pathogen’s proteome using computational tools to identify a subset of protective antigens, commonly referred to as ‘potential vaccine candidates’ (PVCs), as the first stage in vaccine development [9]. The RV field has been in existence for the past 21 years (2000 to 2021). Vaccine design with RV is: (i) relatively quick, as the process takes one to two years to complete; (ii) safe, as the pathogen does not need to be cultured in a laboratory; and (iii) all conceivable PVCs, including those not expressed in vitro, can be found [9]. The detection of PVCs is performed using RV prediction tools (for example, websites or downloadable software) [10]. To this end, VaxiJen [11, 12] is the first RV website founded in the year 2007 and it is the most populous. When a user enters a pathogen’s protein sequence into VaxiJen, the program generates a prediction of whether the sequence is likely to be a protective antigen (PVC) or non-antigen, and a score based on a set of criteria. Many researchers have since used VaxiJen for the in silico prediction of PVC for vaccine design of various pathogens [13–19]. In 2017, Zaharieva et al. [20] published a 10-year review of the PVCs predicted by VaxiJen for different pathogens (including viruses).

However, to the best of our knowledge, there are currently no published reviews of PVCs predicted by VaxiJen for viruses between 2017–2021 in the literature. This research is necessary because (i) it will help vaccine researchers design vaccines for different viruses, (ii) it will allow researchers to undertake in vitro and in vivo experiments to determine whether the PVCs stimulate an immune response, and (iii) the results may aid in identifying gaps for future research. As a result, this protocol proposes a study with the following research objective:

To provide an overview of the potential vaccine candidates predicted by VaxiJen for different viral pathogens between 2017–2021.

A scoping review study will be conducted to achieve this research objective. The scoping review approach was chosen for three reasons: (i) it may provide a wider picture of the topic of interest, serving as precursor to systematic reviews; [21] (ii) this study will not include any clinical questions, which would be more appropriate for a systematic review; [21] and (iii) any research gaps can be identified [21].

## Methods

This protocol is for a scoping review of literature reporting on PVCs predicted by VaxiJen for different viral pathogens between 2017–2021. On February 17, 2022, we registered our protocol with the Open Science Framework (OSF) platform’s registries (registration link: https://osf.io/ht8wr). In addition, we used the Preferred Reporting Items for Systematic Reviews and Meta-Analyses Protocols (PRISMA-P) 2015 checklist [22, 23] (see Additional file 1: Table S1 for the PRISMA-P 2015 Checklist) to report this protocol.

**Fig. 1:**
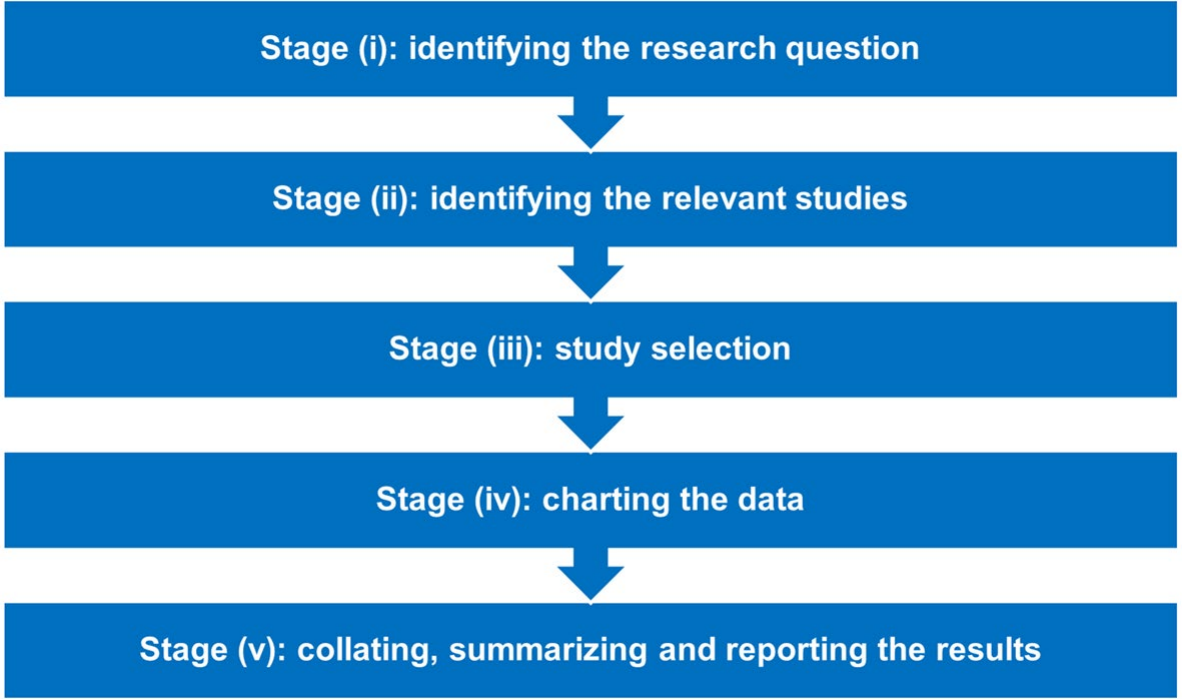
The five key stages of Arksey and O’Malley’s methodological framework for conducting a scoping review

The proposed review will employ the methodological framework by Arksey and O’Malley [24], which was later enhanced by Levac et al. [25] and the Joanna Briggs Institute (JBI) [26]. As seen in Fig. 1, this framework is composed of five fundamental successive stages: (i) identifying the research question, (ii) identifying the relevant studies, (iii) study selection, (iv) charting the data, and (v) collating, summarizing, and reporting the results. These stages are discussed below within the context of the present scoping review.

The abovementioned framework will be used in conjunction with the Preferred Reporting Items for Systematic Reviews extension for Scoping Reviews (PRISMA-ScR) proposed by Tricco et al. [27] PRISMA-ScR provides a reporting guideline containing 20 essential items and two optional items that should be included in scoping reviews [27]. This guideline also facilitates methodological transparency and acceptance of research findings [27].

### Stage (i): identifying the research question

 The research question for this scoping review is: 

What has been reported in the literature regarding potential vaccine candidates predicted by VaxiJen for different viral pathogens between 2017 and 2021?

This study will utilize the population-concept-context (PCC) mnemonic as recommended by the JBI [26] to identify the main elements in the research question (Table 1). This guidance by the PCC will ensure that the study selection is in line with the research question given above.

**Table 1:** The main elements in this study’s research question according to the JBI framework’s PCC mnemonic

**P** **Population**	**C** **Concept**	**C** **Context**
‘different viral pathogens’	‘potential vaccine candidates predicted by VaxiJen’	‘between 2017 and 2021’

*JBI* Joanna Briggs Institute, *PCC* Population-concept-context

### Stage (ii): identifying the relevant studies

A three-step approach will be undertaken for this stage. First, a search will be conducted with the search term ‘vaxijen’ in the following electronic databases: (i) PubMed [28], (ii) Scopus [29], (iii) Web of Science [30], (iv) EBSCOhost [31], and (v) ProQuest One Academic [32] (see Additional file 2: Table S2 for the proposed search strategy per database). The databases listed above are both accessible and relevant to public health, allowing us to compile a comprehensive sample of relevant literature. The eligibility criteria (inclusion and exclusion) will be defined as shown in Table 2. Second, the reference lists from the included papers will be reviewed to identify any additional studies not retrieved by the database searches. A saturation point will be reached when no new sources are identified from the reference lists. The full texts of articles in the reference lists will be reviewed if the first author is unable to decide on the inclusion or exclusion of the study based on title and abstract. Third, the first author will hand-search key journals to identify any articles that may have been missed during database and reference list searches. The first step above is required, whereas the second and third steps will be undertaken only if the total number of articles found from step one is insufficient in scope and breadth.

**Table 2:** Criteria for inclusion and exclusion of articles in this study

**Inclusion criteria**
- Research focused on the usage of VaxiJen for the prediction of PVCs for different viral pathogens - Articles published from the years 2017 to 2021 - Articles written in the English language, as English is the principal investigator’s first language and due to resource limitations - Studies published in peer-reviewed journals and unpublished papers - Articles that have access to the full text - Type of studies: original articles
**Exclusion criteria**
- Research not focused on the usage of VaxiJen for the prediction of PVCs for different viral pathogens - Studies published in the year 2017 that were already covered in Zaharieva et al.’s^20^ review article - Non-English articles will be excluded - Articles without access to the full text - Non-original articles will be omitted

*PVCs* Potential vaccine candidates

### Stage (iii): study selection

The search results from the databases mentioned above will be exported as a.nbib file from PubMed and as a.ris file from the remaining databases. These five exported files will be uploaded to Rayyan [33, 34], an open-source review management software, which will deduplicate the articles. Rayyan supports the.nbib and.ris file formats, and it was chosen to deduplicate articles since it has the maximum sensitivity for reference deduplication [35]. Following deduplication, the remaining publications will be examined in Rayyan by title and abstract (and, if necessary, by browsing through the full text of an article) to identify whether the research fits the inclusion requirements. The full text of the selected articles will be downloaded, screened for eligibility (Table 2) and included. If we are unable to locate the complete text of an article online, we will contact the author(s) to obtain the full text. This screening process will be guided by the main elements in this study’s research question (Table 1). The first author will screen the articles, and the second author will review them. They will resolve any disagreements by discussion until they reach a consensus.

### Stage (iv): charting the data

The fourth step will involve charting the data of selected articles from stage (iii). Arksey and O’Malley [24] suggested that the charting approach must take a broader view and that a common analytical framework should be applied to all selected studies. The ‘descriptive-analytical’ method will therefore be employed in this scoping review [24]. To this end, the first author will develop a data charting form in Microsoft Excel, which will be reviewed by the second author. The charted data will be entered into this data charting form and will include the following fields (Table 3).

**Table 3:** The data charting form for this study

**Proposed fields**
- Pathogen (the name of different viruses) - Year (of publication) - Reference - Key findings (relating to the scoping review question) - Experimentally validated? (this field will be set to ‘yes’ or ‘no’ depending on the results of the study. If ‘yes’, then we will include information about the experimental validations from the study)

### Stage (v): collating, summarizing, and reporting the results

The PRISMA flow diagram [36] (see Additional file 3) will be used to show the number of sources of evidence that were screened, evaluated for eligibility, and included in the review from stage (iii). We will employ the following three distinct stages suggested by Levac et al. [25] to present our results in a rigorous manner: (i) analysing the data, (ii) reporting results, and (iii) applying meaning to the results. First, based on the research objective, research question, and Table 1 of this study, the number of papers identified by (i) year (of publication) and (iii) pathogen (the names of different viruses) will be provided in a line graph and table (see Table 4 for the proposed fields), respectively. Second, in order to achieve the scoping review’s research question and objective, tables will be employed to display the results from the charted data in step (iv) in an ordered manner, as shown in Table 3. In the ‘reference’ field, we shall enter the citation for each paper. Finally, the significance of the study’s findings will be discussed in light of research, policy and practice (experimental validation) to aid us in formulating recommendations.

**Table 4:** Draft template for the number of publications by pathogen

**Proposed fields**
- Pathogen (for example: Ebola virus) - Number of publications (for example: 7)

## Discussion

The aim of the proposed scoping review is to outline the PVCs discovered by VaxiJen for different viral pathogens between 2017–2021. This review will also highlight gaps for further research.

To our knowledge, this study will be the first to review the PVCs predicted by the VaxiJen RV tool for different viruses between 2017–2021. The methodological rigour of the proposed scoping review is its main strength. The PRISMA-P 2015 checklist [22, 23], a scoping review framework proposed by Arksey and O’Malley [24], with enhancements by Levac et al. [25] and the JBI [26] were used to create this scoping review protocol. Similarly, the planned review will be carried out using the framework outlined above, together with Tricco et al.’s [27] PRISMA-ScR reporting guideline. This scoping review protocol was submitted for publication and went through an extensive peer review process. Vaccinology experts may conduct in vitro and in vivo experiments to establish whether the PVCs in the suggested review stimulate any immune response. As a result, the suggested review has implications for the development of innovative vaccines against various viral infections.

The key limitation of this study, on the other hand, will be language bias due to the selection of English-only papers. Despite the fact that VaxiJen can predict PVCs for viruses, bacteria, fungi, parasites, and tumours, the planned scoping review will only look at viruses. Viruses are chosen as the subject of this study since this is also the focus of a broader project. The constraints listed above are due to the project’s limited resources.

This study will solely use secondary data and will not include any patients. As a result, no ethical clearance is necessary. Nevertheless, this study is part of a larger research project that was submitted for ethical consideration to the Biomedical Research Ethics Committee (BREC) of the University of KwaZulu-Natal (UKZN) in Durban, KwaZulu-Natal, South Africa. Any changes to this protocol made over the course of the study will be disclosed in the final paper. This review’s findings will be reported in a manuscript, which will be submitted to an international peer-reviewed journal for publication. In addition, the results will be provided to relevant policymakers, funders, and vaccine researchers. The latter may conduct in vitro and in vivo assays to ascertain the findings reviewed in this study, with the goal of developing efficacious vaccines against different viruses.

## Supplementary Information

**Additional file 1: Table S1.** PRISMA-P 2015 Checklist.

**Additional file 2: Table S2.** Proposed search strategy per database.

**Additional file 3.** PRISMA 2020 flow diagram for new systematic reviews which included searches of databases and registers only.

## Data Availability

Not applicable.

## Abbreviations

BREC: Biomedical Research Ethics Committee
HIV: Human immunodeficiency virus
JBI: Joanna Briggs Institute
OSF: Open Science Framework
PCC: Population-concept-context
PRISMA-P: Preferred Reporting Items for Systematic Reviews and Meta-Analyses Protocols
PRISMA-ScR: Preferred Reporting Items for Systematic Reviews extension for Scoping Reviews
PVCs: Potential vaccine candidates
RV: Reverse vaccinology
UKZN: University of KwaZulu-Natal
WHO: World Health Organization
