# Supplementary material for: Protocol for a scoping review of potential vaccine candidates predicted by VaxiJen for different viral pathogens between 2017–2021
Source: Syst Rev. 2022 Dec 30;11:284. doi: 10.1186/s13643-022-02121-0 (PMC9801145; doi:10.1186/s13643-022-02121-0)
Supplement: Supplementary file 2 — Additional file 2: Table S2. Proposed search strategy per database. [file 13643_2022_2121_MOESM2_ESM.docx]

**Additional file 2**

**Table S2: Proposed search strategy per database**

| Database | Search strategy |
| --- | --- |
| PubMed | Search term: "vaxijen"  Language: English  Publication date: from 2017 to 2021 |
| Scopus | ALL ("vaxijen") AND (LIMIT-TO (PUBYEAR, 2021) OR LIMIT-TO (PUBYEAR, 2020) OR LIMIT-TO (PUBYEAR, 2019) OR LIMIT-TO (PUBYEAR, 2018) OR LIMIT-TO (PUBYEAR, 2017)) AND (LIMIT-TO (DOCTYPE, "ar")) AND (LIMIT-TO (LANGUAGE, "English")) |
| Web of Science | (((ALL=("vaxijen")) AND PY=(2017-2021)) AND DT=(Article)) AND LA=(English) |
| EBSCOhost | "vaxijen" (All Fields)  Language: English  Published date: start year: 2017; end year: 2021  Publication type: academic journal, primary source document, working paper  Document type: article, journal article, research, working paper |
| ProQuest One Academic | "vaxijen" (Anywhere - all fields)  Language: English  Date: from 2017 to 2021  Document type: article, working paper/pre-print |
