# Supplementary material for: Protocol for a scoping review of potential vaccine candidates predicted by VaxiJen for different viral pathogens between 2017–2021
Source: Syst Rev. 2022 Dec 30;11:284. doi: 10.1186/s13643-022-02121-0 (PMC9801145; doi:10.1186/s13643-022-02121-0)
Supplement: Supplementary file 3 — Additional file 3. PRISMA 2020 flow diagram for new systematic reviews which included searches of databases and registers only. [file 13643_2022_2121_MOESM3_ESM.docx]

**Identification of studies via databases and registers**

Records removed *before screening*:

Duplicate records removed (n = )

Records marked as ineligible by automation tools (n = )

Records removed for other reasons (n = )

Records identified from*:

Databases (n = )

Registers (n = )

**Identification**

Records screened

(n = )

Records excluded**

(n = )

Reports sought for retrieval

(n = )

Reports not retrieved

(n = )

**Screening**

Reports assessed for eligibility

(n = )

Reports excluded:

Reason 1 (n = )

Reason 2 (n = )

Reason 3 (n = )

etc.

Studies included in review

(n = )

Reports of included studies

(n = )

**Included**

*Consider, if feasible to do so, reporting the number of records identified from each database or register searched (rather than the total number across all databases/registers).

**If automation tools were used, indicate how many records were excluded by a human and how many were excluded by automation tools.

*From:*  Page MJ, McKenzie JE, Bossuyt PM, Boutron I, Hoffmann TC, Mulrow CD, et al. The PRISMA 2020 statement: an updated guideline for reporting systematic reviews. BMJ 2021;372:n71. doi: 10.1136/bmj.n71

For more information, visit: <http://www.prisma-statement.org/>
